# Supplementary material for: Is Shiga Toxin-Producing Escherichia coli O45 No Longer a Food Safety Threat? The Danger is Still Out There
Source: Microorganisms. 2020 May 22;8(5):782. doi: 10.3390/microorganisms8050782 (PMC7285328; doi:10.3390/microorganisms8050782)
Supplement: Supplementary file 1 [file microorganisms-08-00782-s001.pdf]

Supplementary Material

Table S1 Information of reference Shiga toxin-producing *E. coli* genomes obtained from NCBI in this study.

Strain ID	Serotype	Isolation Source	Isolation Country	GenBank accession nubmer	
				Chromosome	Plasmid
11368	O26:H11	Outbreak strain	Japan	AP010953	AP010954, AP010955
FWSEC0003	O45:H2	Clinical strain	Canada$^{\alpha}$	CP031916	CP031917, CP031918
2011C-4251	O45:H2	Clinical strain	USA	CP027388	CP027389
12009	O103:H2	Clinical strain	Japan	AP010958	AP010959
2015C-3163	O103:H2	Clinical strain	USA	CP027219	CP027220
2013C-4225	O103:H11	Clinical strain	USA	CP027578	CP027578
2013C-3264	O103:H25	Outbreak strain	USA	CP027544	CP027545
2011C-3493	O104:H4	Outbreak strain	USA	CP00328	CP003290,CP003291
16-9255	O121:H19	Outbreak strain	Canada$^{\beta}$	CP022407	CP022408
RM13514	O145:H28	Outbreak strain	USA	CP006027	CP006028, CP006029
Sakai	O157:H7	Outbreak strain	Japan	BA000007	AP018692, AB011548

$^{\alpha}$ Strain FWSEC0003 was isolated from Halifax, Canada; $^{\beta}$ Strain 16-9255 was isolated from Saskatchewan, Canada.
